# The Characterization of Feces and Urine: A Review of the Literature to Inform Advanced Treatment Technology

**DOI:** 10.1080/10643389.2014.1000761

**Published:** 2015-05-29

**Authors:** C. Rose, A. Parker, B. Jefferson, E. Cartmell

**Affiliations:** ^a^ Cranfield Water Science Institute, Cranfield University, Cranfield, Bedfordshire, United Kingdom

**Keywords:** fecal characteristics, feces, feces treatment, human excreta, urine, urine characteristics

## Abstract

The safe disposal of human excreta is of paramount importance for the health and welfare of populations living in low income countries as well as the prevention of pollution to the surrounding environment. On-site sanitation (OSS) systems are the most numerous means of treating excreta in low income countries, these facilities aim at treating human waste at source and can provide a hygienic and affordable method of waste disposal. However, current OSS systems need improvement and require further research and development. Development of OSS facilities that treat excreta at, or close to, its source require knowledge of the waste stream entering the system. Data regarding the generation rate and the chemical and physical composition of fresh feces and urine was collected from the medical literature as well as the treatability sector. The data were summarized and statistical analysis was used to quantify the major factors that were a significant cause of variability. The impact of this data on biological processes, thermal processes, physical separators, and chemical processes was then assessed. Results showed that the median fecal wet mass production was 128 g/cap/day, with a median dry mass of 29 g/cap/day. Fecal output in healthy individuals was 1.20 defecations per 24 hr period and the main factor affecting fecal mass was the fiber intake of the population. Fecal wet mass values were increased by a factor of 2 in low income countries (high fiber intakes) in comparison to values found in high income countries (low fiber intakes). Feces had a median pH of 6.64 and were composed of 74.6% water. Bacterial biomass is the major component (25–54% of dry solids) of the organic fraction of the feces. Undigested carbohydrate, fiber, protein, and fat comprise the remainder and the amounts depend on diet and diarrhea prevalence in the population. The inorganic component of the feces is primarily undigested dietary elements that also depend on dietary supply. Median urine generation rates were 1.42 L/cap/day with a dry solids content of 59 g/cap/day. Variation in the volume and composition of urine is caused by differences in physical exertion, environmental conditions, as well as water, salt, and high protein intakes. Urine has a pH 6.2 and contains the largest fractions of nitrogen, phosphorus, and potassium released from the body. The urinary excretion of nitrogen was significant (10.98 g/cap/day) with urea the most predominant constituent making up over 50% of total organic solids. The dietary intake of food and fluid is the major cause of variation in both the fecal and urine composition and these variables should always be considered if the generation rate, physical, and chemical composition of feces and urine is to be accurately predicted.

## 1. INTRODUCTION

An estimated 2.6 billion people in the world lack access to improved sanitation, defined as the hygienic separation of human excreta from human contact (WHO/UNICEF, 2012). Diseases that are associated with inadequate sanitation are particularly associated with poverty and account for 10% of the total disease burden worldwide (Prüss-Üstün et al., 2008). Poor sanitation and fecal sludge management not only have negative impacts on human health but also affect the environment through the contamination of water bodies, soils, and food sources (Peletz et al., 2011; Ziegelbauer et al., 2012). In 2010, 72% of sanitation facilities in Sub-Saharan Africa and 59% in Southern Asia were classified as “unimproved” (WHO/UNICEF, 2012). On-site sanitation (OSS) facilities are the predominant form of excreta disposal in urban populations of low income areas; for example, in urban areas of Ghana and Tanzania 85% of inhabitants are served by OSS facilities and in urban areas of the Philippines 98% rely on OSS facilities (Montangero and Strauss, 2004). However, when these facilities need emptying, there are often inadequate facilities or financial disincentives for the proper disposal of fecal sludge meaning that pits remain full and unusable or if emptied, sludge is disposed of directly into the environment contaminating water resources (Ingallinella et al., 2002). This problem has inspired the development of OSS technologies that treat excreta directly at or close to its source, producing safe and beneficial products with no need for further transport. This factor is illustrated by a rapid rise in research and development in OSS technology, with the Bill and Melinda Gates Foundation (BMGF) funding 16 “Reinvent the Toilet Challenge” (RTTC) research projects worldwide since 2011, with the second round of grants totaling nearly US$3.4 million in 2012 (Global Development Program, 2014). This trend is continuing with the BMGF investing in regional programs, for example, US$5 million has been awarded to Chinese research institutes to drive research and development into new OSS systems (Global Development Program, 2014).

Knowledge of the waste that enters treatment systems is a basic prerequisite for the design and development of future technology. There is information on conventional sanitary sewage (Henze et al., 2001; Tchobanoglous et al., 2003) but this material has a different composition to fresh feces and urine which has not undergone any degradation processes and will have substantially less water or gray water addition. Instead generation rates and the chemical composition of feces and urine in the human population are key factors to be understood by OSS technology developers. A number of medical studies have determined the fecal and urine output of human populations, however the data are specific to distinct populations defined by geography, age, ethnicity, disease, and diet. There have so far been no attempts to summarize these data and understand the major causes of variation. The aim of this study is to review the variation, generation rate, and chemical and physical composition of the solid and liquid fractions of human excreta that would supply OSS technologies in developing countries. An assessment will then be made on how the results and any variation found will impact on potential treatment technology.

## 2. METHODS

Generation rate, composition, and physical and chemical nature of both feces and urine were recorded as of Table 1. Each recorded datum was the mean of the data from the reported study. Some published papers reported two or more independent studies so these papers contributed more than one value to the data set. The mean and median of each variable were both calculated as measures of central tendency and data were checked for normality by calculating a coefficient of skewness (Young, 1962):
(1) 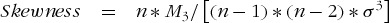

(2) 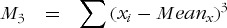

**Table 1:** Measured variables for feces and urine

	Feces unit	Urine unit
Variable	of measure	of measure
*Generation*	g/cap/day	L/cap/day
Frequency of defecation	motions/24 hr	urinations/24 hr
Water content	% total mass	% total mass
*Organic composition*	% total mass	% dry mass
Components of solids	% total mass	% total mass
*Inorganic composition*	% dry mass	% dry mass
Daily excretion of elements	g/cap/day	g/cap/day, mg/L
*Chemical nature*		
pH	pH	pH
COD and BOD	mg/g wet mass	mg/L
*Physical form*		
Bristol stool form	Linear scale (1–7)	
Diarrhea prevalence	% of population	

*σ = Standard deviation*

*n = Valid number of cases*

Box and whisker plots were created using Statistica 11 software (Statsoft Inc., Tulsa, OK, USA, 2011). Outliers of each data set were defined using a standard default outlier coefficient value (Burns et al., 2005).
(3) 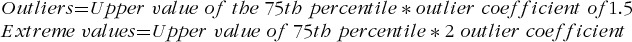



No outliers were removed from the data set but were identified in the graphical output. Full statistical calculations were only conducted on variables that had at least seven values but a median value is given for data when there were less than seven values.

A summary of studies used in the statistical analysis are outlined in Table 2, including the location and number of studies. A large proportion (80%) of the data set was from studies conducted in Europe and North America. A distinction was therefore made between low and high income countries by the measure of development; using the Human Development Index (HDI), a composite index measuring average achievement in three basic dimensions of human development; life expectancy, education, and income (UNDP, 2011).

**Table 2:** The geographical location and human development index ranking of studies used in statistical analysis

Country	n	HDI*	References
Africa	2	3/4^a^	Cranston and Burkitt (1975), Burkitt et al. (1980)
Australia	2	1	Birkett et al. (1996), Hovey et al. (2003)
Burma	1	4	Myo-Kin et al. (1994)
Canada	3	1	Burkitt et al. (1980), Vuksan et al. (1999)
China	3	2	Jie et al. (2000), Chen et al. (2008), Bai and Wang (2010)
Denmark	2	1	Maclennan and Jensen (1977), Jensen et al. (1982)
Developing countries	2	3/4^a^	Feachem et al. (1978)
Europe and North America	1	1/2^b^	Feachem et al. (1978)
European	1	1^b^	Mykkänen et al. (1998)
Finland	4	1	Reddy et al. (1975), Reddy et al. (1978), Jensen et al. (1982), Mykkänen et al. (1998)
Germany	1	1	Erhardt et al. (1997)
Guatemala	1	3	Calloway and Kretsch (1978)
Holland	4	1	Stasse-Wolthuis et al. (1980), Van Faassen et al. (1993), Gaillard (2002), Wierdsma et al. (2011)
India	1	3	Shetty and Kurpad (1986)
Iran	1	2	Adibi et al. (2007)
Japan	7	1	Glober et al. (1977), Polprasert and Valencia (1981), Tarida et al. (1984), Saitoh et al. (1999), Danjo et al. (2008), Shinohara et al. (2010), Hotta and Funamizu (2009)
Kenya	1	4	Cranston and Burkitt (1975)
New Zealand	1	1	Pomare et al. (1981)
North America	1	1^b^	Vuksan et al. (2008)
Peru	1	2	Crofts (1975)
Singapore	1	1	Chen et al. (2000)
South Africa	2	3	Burkitt et al. (1972), Walker (1975)
Spain	1	1	Roig et al. (1993)
Sweden	4	1	Reddy et al. (1978), Vinneras (2002), Vinnerås et al. (2006)
Thailand	2	2	Danivat et al. (1988), Schouw et al. (2002)
Tonga	1	2	Pomare et al. (1981)
UK	26	1	Olmsted et al. (1934), Connell et al. (1965), Southgate and Durnin (1970), Burkitt et al. (1972), Goy et al. (1976), Wyman et al. (1978), Prynne and Southgate (1979), Stephen and Cummings (1980), Eastwood et al. (1984), Eastwood et al. (1986), Davies et al. (1986), Cummings et al. (1987), Sandler and Drossman (1987), Cummings et al. (1992), Murphy et al. (1993), Cummings et al. (1996), Lewis and Heaton (1997), Chen et al. (1998), Reddy et al. (1998), Rivero-Marcotegui et al. (1998), Aichbichler et al. (1998), Almeida et al. (1999), Magee et al. (2000), Chaplin et al. (2000), Woodmansey et al. (2004), Silvester et al. (2011)
USA	18	1	Canfield et al. (1963), Watts et al. (1963), Diem and Lentner (1970), Goldsmith and Burkitt (1975), Cummings et al. (1978), Glober et al. (1977), Goldberg et al. (1977), Beyer and Flynn (1978), Reddy et al. (1978), Calloway and Kretsch (1978), Kien et al. (1981), Polprasert and Valencia (1981), Tucker et al. (1981), Schubert et al. (1984), Parker and Gallagher (1988), Zuckerman, et al. (1995), Aichbichler et al. (1998), McRorie et al. (2000)

*Human Development Index Classifications (UNDP, 2011): 1. Very high, 2. High, 3. Medium, 4. Low.^a^Classification not available, presumed to be ranking 3 or 4.^b^Classification not available, presumed to be ranking 1 or 2.

Preliminary data analysis indicated that fiber intake was a major cause of variation in fecal generation and composition. There were a sufficient number of studies that had examined the effects of fiber intake on fecal output to enable further analysis to be undertaken on these data. The total dietary fiber intake was related to the generation of feces in linear and nonlinear regression analyses.

## 3. RESULTS

### 3.1 Feces Generation

**Figure 1:**
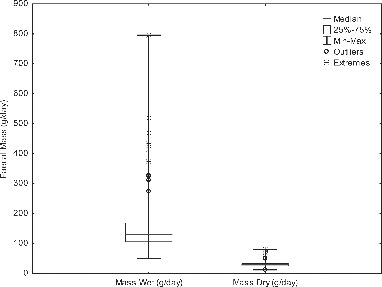
Daily wet and dry mass of feces produced by human populations (g/cap/day). Outliers represent the upper value of the 75th percentile multiplied by the outlier coefficient (1.5), (extreme values = upper value of 75th percentile *2 outlier coefficient). Fecal wet mass generation (n = 112) has a large range and was an abnormal data set. Fecal dry mass (n = 61) showed a smaller range with fewer outliers and extreme values.

Fecal wet mass values have a median figure of 128 g/cap/day. This is from a distribution of 116 mean values from studies reporting healthy individuals, with a large minimum and maximum range of 51–796 g/cap/day (Figure 1). However, as mean values for each study were recorded, individual variation within these studies is not accounted for; if all values are recorded the range extends to 15–1505 g/cap/day. The data set for mean wet fecal generation had a positive skew, hence the mean was greater than the median. The low income countries data set was not as skewed as the high income countries (Table 3)[Table T0004] [Table T0005]. This is likely a result of the wider range of diets that can be consumed by populations in richer countries. A statistically significant difference (t = 2.87, *p* <.05) between mean values of high income countries and low income countries was found in regards to wet fecal weight. As a collective group high income countries had relatively small per capita wet fecal weights in comparison to low income countries. However, between individual studies there was a large variation of 51–796 g/cap/day, despite all studies reporting healthy individuals. For low income countries the median value of 250 g/cap/day was larger in comparison to the median value of 126 g/cap/day in high income countries.

**Table 3:** Daily wet and dry mass produced by humans from low and high income populations

	Wet weight	Wet weight	Dry weight	Dry weight
	(g/cap/day)	(g/cap/day)	(g/cap/day)	(g/cap/day)
	High income*	Low income*	High income*	Low income*
Median	126	250	28	38
n	95	17	57	8
Minimum	51	75	12	18
Maximum	796	520	81	62
Skewness	4.178	0.598	2.378	0.098
Std. error of skewness	0.248	0.550	0.327	0.752
Mean	149	243	30	39
St dev	95.0	130.2	11.7	14.1
Variance	9024	16,960	136	201

*Classifications acquired from the 2011 HDI report (UNDP, 2011) where the four tiers were split into two sections with “very high” and “high” comprising the high income classification and “medium” and “low” comprising the low income classification.

**Table 4 T0004:** The effect of diet type on fecal characteristics

	Fiber	Number	Fecal	Fecal	Stool			
Diet	intake	of subjects	mass	mass	frequency	Moisture	Fecal	
type*	(g/day)	in study	wet (g/day)	dry (g/day)	(motions per 24 hr)	(%)	pH	References
Omnivore	23	17	153		1			Davies et al. (1986)
Vegetarian	37	17	168		1.2			Davies et al. (1986)
Vegan	47	17	225		1.7			Davies et al. (1986)
Omnivore		14			1.4	73.5		Goldberg et al. (1977)
Vegetarian		14			1.8	73.3		Goldberg et al. (1977)
Omnivore		66	131.9					Lewis and Heaton (1997)
Omnivore	16.6	22	117	30.8		72.6	6.65	Reddy et al. (1998)
Vegetarian	16.2	22	186	36		78.9	6.18	Reddy et al. (1998)
Vegetarian	29.3	18	160	38.4		74.6	6.55	Reddy et al. (1998)
Omnivore^a^	12	8	129	32.8		74	7	Silvester et al. (1997)
Omnivore^b^	11	8	118	32		70.7	7.2	Silvester et al. (1997)
Omnivore	27.3	149	119	27.1	0.9		6.8	Van Faassen et al. (1993)
Vegetarian	40.8	11	189	27.9	1.5		6.8	Van Faassen et al. (1993)

*O: Omnivore, V: Vegetarian, VN: Vegan.^a^Low meat diet (68 g/day protein).^b^High meat diet (192 g/day protein).

**Table 5 T0005:** Daily loadings and concentrations of elements in feces (wet weight)

	Value	Value	
	(g/cap/day)	(g/kg)	References
Total P	0.35	3.40	Vinnerås et al. (2006)
	0.5	1.83	Czemiel (2000)
	0.5	3.59	Vinneras (2002)
	0.51	1.77	Goldblith and Wick (1961)
	0.65–0.87	7.76–8.92	Calloway and Margen (1971)
	0.5	3.8	Meinzinger and Oldenburg (2009)
	0.69–2.5	4.80–9.86	Chaggu (2004)
	0.9–2.7		Wignarajah et al. (2003)
Total K	0.20–0.24	1.78–2.14	Calloway and Margen (1971)
	0.47	3.10	Goldblith and Wick (1961)
	0.75–0.88		Wignarajah et al. (2003)
	0.8	4.936	Eastwood et al. (1984)
	0.8–1.0		Kujawa-Roeleveld and Zeeman (2006)
	0.7	3.3	Meinzinger and Oldenburg (2009)
	0.8–2.1	2.712	Chaggu (2004)
	1.48–2.52	7.16	Vinnerås et al. (2006)
Na	0.12	0.80	Goldblith and Wick (1961)
	0.8 (0.3–4.1)	4.94	Eastwood et al. (1984)
Ca	0.1–1		Wignarajah et al. (2003)
	2.9–3.6		Chaggu (2004)
	0.53		Kujawa-Roeleveld and Zeeman (2006)
	0.61	3.77	Eastwood et al. (1984)
	0.64	4.27	Goldblith and Wick (1961)
	0.96–1.12	2.68	Calloway and Margen (1971)
Mg	0.15	0.93	Eastwood et al. (1984)
	0.18		Kujawa-Roeleveld and Zeeman (2006)
	0.20	1.33	Goldblith and Wick (1961)
	0.30–0.34	2.86	Calloway and Margen (1971)
Cl	0.09	0.6	Goldblith and Wick (1961)
S	0.13	0.87	Goldblith and Wick (1961)
	0.2		Meinzinger and Oldenburg (2009)
	(mg/cap/day)	(mg/kg)	
Cu	1.02	6.8	Goldblith and Wick (1961)
	1.10		Kujawa-Roeleveld and Zeeman (2006)
	1.5–2.1		Wignarajah et al. (2003)
Fe	30	200	Goldblith and Wick (1961)
	700–1000		Wignarajah et al. (2003)
Pb	0.03–0.07	0.12–0.27	Schouw et al. (2002)
	0.02–0.03		Hansen and Tjell (1979)
	1.26	6.38	Vinnerås et al. (2006)
Mn	24–90		Wignarajah et al. (2003)
Mo	2–4		Wignarajah et al. (2003)
Zn	7.85	48.46	Eastwood et al. (1984)
	5–10		Wignarajah et al. (2003)
	10.68		Kujawa-Roeleveld and Zeeman (2006)
	13.31	67.49	Vinnerås et al. (2006)
Ni	0.08–0.09		Hansen and Tjell (1979)
	0.3	1.52	Vinnerås et al. (2006)
	0.3	1.15	Schouw et al. (2002)
Cr	0.02–0.03		Hansen and Tjell (1979)
	0.08	0.31	Schouw et al. (2002)
	0.18	0.91	Vinnerås et al. (2006)
Cd	0.07	0.27	Schouw et al. (2002)
	1.26	6.39	Vinnerås et al. (2006)
Hg	0.007	0.04	Vinnerås et al. (2006)

The mean weight of children's feces (3–18 years) has been recorded between 75 and 374 g/cap/day (Burkitt et al., 1972; Tandon and Tandon, 1975; Burkitt et al., 1980; Almeida et al., 1999; Schouw et al., 2002). Infants (1–4 years) were shown to have a mean stool weight of 85 g/cap/day with no significant difference found between the age of children in years, however, a weak correlation was found between the infants age in months and total stool weight (r = 0.125, *p* <.029) (Myo-Khin et al., 1994). Mean values for elderly subjects (aged 65 years or more) were reported at 158 g/cap/day by Mykkänen et al. (1998) and 70 g/cap/day by Woodmansey et al. (2004).

Median dry stool weight was 29 g/cap/day which were recorded from the mean values of 60 studies, with a range of means of 12–81 g/cap/day (Figure 1). Again, individual variation within these studies was not accounted for as mean values of these populations were taken; ranges of minimum and maximum values taking into account individual variation within these studies was subsequently larger at 4–102 g/cap/day dry solids. The data set was not of a normal distribution with a positive skew of 1.8. This was also due to the skewed distribution of values from high income countries (Table 3). The median dry weight of feces is 25% of the wet weight of feces (n = 45) with values in the range of 11–34% reported (Figure 1).

#### 3.1.1 Factors Affecting Fecal Mass

The major factors leading to variation in fecal generation rate are total food intake, body weight, and diet. Parker and Gallagher (1992) found that mean daily stool weight was correlated (*p* <.001) with calorie intake (energy intake can act as a measure of food intake); however, they found that this only accounted for 28% of the variation seen in individual stool output. Body weight also represents differing energy intake requirements; for example, as a guideline a healthy adult requires 20–25 kcal/per kilogram of body weight (Moyes and McKee, 2008). The increasing body weight therefore reflects increasing energy intake which in turn can act as a measure of total food intake. Food intake and body weight therefore have an influence over fecal weight and this accounts for variables such as gender (Stephen et al., 1986; Lampe et al., 1993; Poullis et al., 2004) and race (Burkitt et al., 1972; Goldsmith and Burkitt, 1975) that have been observed as being significant within the literature.

**Figure 2:**
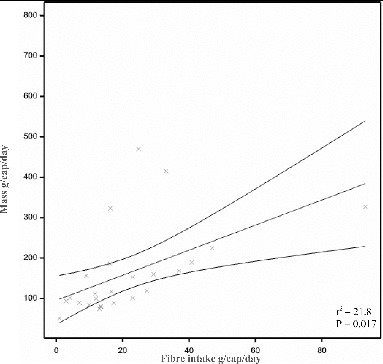
Fitted and observed relationship with 95% confidence limits. Values from 22 studies where fiber intake was recorded. Three large outliers were recorded, however, no reason could be found to exclude these results from the study. There was a significant correlation between dietary fiber intake and fecal output (r^2^ = 21.8, *p* =.017) with an intercept 101.3 ± 34.3 and a regression coefficient of 2.96 ± 1.13.

Human diet is also a factor that can impact the generation rate and composition of feces (Table 4). Fiber intake is often cited for causing variation in feces production, for example, by Vuksan et al. (2008). Regression analysis of secondary data presented in 25 studies where fiber intake was recorded was conducted and results show that fecal wet mass was positively correlated with fiber intake (r = 2.96 ± 1.13, *p* =.017) (Figure 2).

The effect of dietary fiber on fecal weight is highly dependent upon the type of fiber consumed (non-degradable or degradable). Non-degradable fiber undergoes minimal changes in the digestive tract as it is relatively un-fermentable and shortens colonic transit time (Bijkerk et al., 2004); wet fecal mass has been negatively correlated with transit time, r = −0.22, *p* <.05 (Eastwood et al., 1984). Non-degradable fiber has a high water holding capacity which promotes bulk and increased defecation frequency; extensive studies with non-degradable cereal fibers have shown this (Cummings et al., 1992; Hughes et al., 2002; Vuksan et al., 2008). In a study on wheat bran by Vuksan et al. (2008) a ratio of 2.8 g stool/per g additional fiber on top of a control diet was observed. Degradable fibers can also cause an increase in fecal mass. Highly degradable types of fiber (such as cabbage fiber or oat bran) are fermented in the colon by bacteria much more than non-degradable fibers (Bijkerk et al., 2004). However, degradable fibers still increase fecal weights due to the proliferation of the bacterial component that is stimulated by the presence of a fermentable substrate (Garrow et al., 1993); the resultant increase in bacterial mass is soft, bulky, and water retaining (FAO/WHO, 1997). Any alteration in the bacterial biomass component is significant as it can make up to 55% of total fecal solids (Stephen and Cummings, 1980). Therefore, the impact of dietary fiber on increasing fecal mass is dependent on the type of fiber consumed.

Polysacharides such as resistant starches (RS) have similar properties to fiber and have also been shown to increase fecal wet weight in many studies (Shetty and Kurpad, 1986; Cummings et al., 1996; Silvester et al., 1997). Diets high in RS have shown a significant increase in fecal wet and dry weight; (Phillips et al., 1995) concluded that for every 1 g RS consumed (mean 34 g/day) there was an increase in the fecal wet weight of 1.8 g. Undigested starch, as measured by dietary intake, reaching the colon was found to increase fecal output (g wet weight/day) by 42% (Phillips et al., 1995). This correlation can be largely attributed to increases in bacterial biomass with fermentation (Cummings et al., 1996).

#### 3.1.2 Stool Frequency

**Figure 3:**
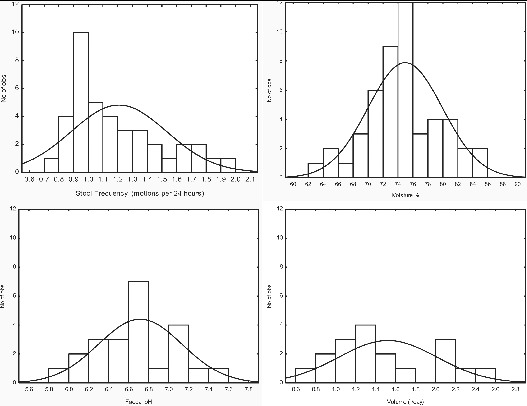
*Top left:* Mean stool frequency in healthy subjects from a wide range of studies (n = 39). Ranges of individuals within these studies varied from 0.21 to 2.54 motions per 24 hr. *Top right:* Mean moisture composition of feces (n = 47). *Bottom left:* Mean fecal pH values from a range of studies (n = 28) consuming a variety of different diets. *Bottom right:* Mean volume of total urine excreted (n = 14).

Defecation frequency provides an indication for design parameters relating to treatability as it provides an indication of how often a facility may be used. Stool frequency also provides an indication of the resultant texture and form of the fecal matter (see physical form section). Mean stool frequency across studies (n = 39) ranged from 0.74 to 1.97 motions per 24 hr with a median value of 1.10 motions per 24 hr period (Figure 3). This represents a guideline figure for a population majority, however, within this variability exists. In a study by Parker and Gallagher (1988) of over 25,000 days worth of data, individuals had a range of means between 0.21 and 2.54 movements per 24 hr illustrating the variability that can occur for individuals in the same population. In a study of a UK population defecations were recorded per hour of the day; the majority of defecations, 61% and 59% in men and women respectively occurred in the morning (06:00–10:00) with peak times in men (20%) occurring between 07:00 and 08:00 and an hour later in women (21%) (Heaton et al., 1992). Another small peak in defecation timing was recorded at 17:00 and 18:00 which is a common time for the evening meal and few defecations were recorded during the night (01:00 to 05:00) (Heaton et al., 1992). The increase in defecation after meal times is primarily due to the resultant increased motor activity of the colon (Christensen, 1985).

Stool frequency is impacted by an individual's health (see physical form section) as well as their fiber intake which is associated with more rapid transit times (Gear et al., 1981). Fiber intake has been positively correlated with stool frequency (r = 0.8, *p* <.001 wet weight; r = 0.5, *p* =.008 dry weight) (Southgate et al., 1976). The inclusion of fiber from fruit and vegetables in the diet has been proven to decrease transit time (*p* <.05) and increase the number of defecations (*p* <.001) (Kelsay et al., 1978). For instance, in a study by Vuksan et al. (2008) high fiber breakfast cereals induced a shorter intestinal transit time and an increased stool frequency. In a meta-analysis of five relevant randomized controlled trials by Yang et al. (2012) dietary fiber was proven to increase stool frequency (odds ratio = 1.19; 95% CI: 0.58–1.80, *p* <.05).

Amongst adults no consistent relationship between frequency of defecation and age was observed (Heaton et al., 1992). Similarly amongst infants there was no significant difference in frequency of defecation between different age categories (Myo-Khin et al., 1994). A lower defecation frequency has been observed in females than in males (Van Faassen et al., 1993; Zuckerman et al., 1995; Chen et al., 2000) and this was accounted for by the longer intestinal transit time of females (*p* <.02) (Gear et al., 1981). However, in children no significant difference was observed between the defecation frequency of boys (0.99/24 hr) and girls (0.96/24 hr) (Myo-Khin et al., 1994). A study by Sandler and Drossman (1987) undertaken in the USA, indicated that the daily mean number of stools varied by race and by sex; whites had more frequent stools than non-whites at 1.3 versus 0.86 defecations/24 hr respectively and men had more frequent stools than women at 1.31 versus 0.96 defecations/24 hr respectively. Conversely, in a study of an Iranian population by Adibi et al. (2007) men were reported to have fewer bowel frequencies per day (1.78 versus 1.97).

### 3.2 Composition

Feces are composed of water, protein, undigested fats, polysaccharides, bacterial biomass, ash, and undigested food residues. The major elements in feces as a percentage of wet weight are oxygen 74%, hydrogen 10%, carbon 5%, and nitrogen 0.7%, including the hydrogen and oxygen present in the water fraction of the feces (Snyder et al., 1975).

Feces compose a median value of 75% H_2_O (n = 47) with a range of 63–86% across mean values of studies (Figure 3), variation can be attributed to differences in fiber intake as non-degradable fiber absorbs more water in the colon (Eastwood, 1973); therefore, as shown in a study by Reddy et al. (1998) those with vegetarian diets will have a higher moisture content of 78.9% whereas those who consume less fiber and more protein will have a lower moisture content of 72.6% (*p* =.001). Fiber intake also affects transit time, which has been positively correlated (r = 0.4, *p* =.03) with% dry matter (Silvester et al., 1997), showing the shorter the intestinal transit time the higher the water content. Variation in moisture content has been shown to vary with age; elderly people were found to excrete the highest amount of water in excreta of all age groups by Schouw et al. (2002). Further deviations from the median value can be caused by illness (see physical composition section). The mean generation rate of fecal water (n = 47) is 0.1 L/cap/day. Average pH values for fecal water have been recorded at pH 6.9 with a range of pH 5.0–8.0 (Mai et al., 2009).

**Figure 4:**
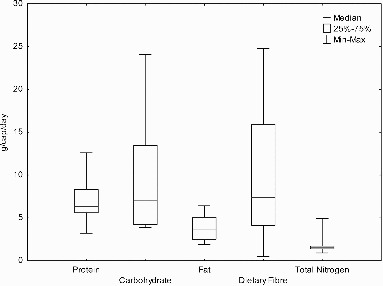
Daily per capita weights of organic fractions excreted in feces.

#### 3.2.1 Organic Fraction

The remaining 25% of feces is therefore composed of solid material. Of the solid fraction organic material makes up between 84% and 93% (Feachem et al., 1978; Nwaneri et al., 2008; Bai and Wang, 2011). The organic solids fraction can be further broken down to the fractions of 25–54% bacterial biomass (Stephen and Cummings, 1980; Guyton and Hall, 2000), 2–25% protein or nitrogenous matter (in addition 50% of bacterial biomass is protein) (Canfield et al., 1963; Volk and Rummel, 1987), 25% carbohydrate or any other nonnitrogenous undigested plant matter (Volk and Rummel, 1987), and 2–15% undigested lipids (Kien et al., 1981; Chen et al., 1998; Wierdsma et al., 2011). These fractions are highly dependent on dietary intake and its biological availability.

The organic fraction therefore makes up the majority of dried solids. Carbon content of feces is between 44% and 55% of dried solids (Feachem et al., 1978; Strauss, 1985) or 7 g/cap/day (Snyder et al., 1975). Volatile solids were shown to comprise 92% of the total solids (TS) fraction of feces (Fry and Merrill, 1973). The bulk organic content of feces can also be measured by chemical oxygen demand (COD) and biological oxygen demand (BOD) values (Table 6). Per capita daily values for BOD were between 14 and 33.5 g/cap/day. Values of COD were measured between 46 and 96 g/cap/day or 567 and 1671 mg/g dry fecal sample. Gas production of human feces was placed at 0.02–0.28 per kg wet feces (United Nations, 1984).

**Table 6:** Loading rates and concentration of BOD and COD in feces

BOD	COD	COD	COD	COD	
(g/cap/day)	(g/cap/_day)	(mg/L)	(mg/g dry)	(mg/g wet)	References
1223*	1668*				Vinnerås et al. (2006)
		48,900			Takahashi et al. (1989)
			1450		Lopez Zavala et al. (2002)
			1380		Almeida et al. (1999)
			1130		Nwaneri et al. (2008)
45					Heinss et al. (1998)
14–34	46–55				Kujawa-Roeleveld and Zeeman (2006)
			567		Chaggu et al. (2007)
			1671		Bai and Wang (2010)
38	96				Choi et al. (2004)
19.3					Fourie and Van Ryneveld (1995)
			1448	354	Buckley et al. (2008)
32	50				Meinzinger and Oldenburg (2009)
		46,230–78,310			Chaggu (2004)

*Includes toilet paper.

#### 3.2.2 Bacterial Composition

A significant proportion of fecal mass consists of bacteria with estimates of combined dead and living bacteria of approximately 25–54% of dry solids (Stephen and Cummings, 1980; Guyton and Hall, 2000; Achour et al., 2006). The wide variation observed is due to differing methodology used between microscopic counting techniques and the separating of bacterial biomass. The high nitrogen content of feces is partly due to undigested protein voided in the feces but is also due to the significant protein content of bacterial biomass in the feces, a figure of 50% protein was proposed by Volk and Rummel (1987); however, a more precise figure is not possible to determine due to uncertainties in the total bacterial composition of feces. A detailed break down of the microbial composition of feces has been compiled by Stephen and Cummings (1980).

#### 3.2.3 Nitrogen/Protein

Nitrogen voided in feces is also recorded as protein. The protein content of feces can be estimated by multiplying the determined nitrogen content by a nitrogen-to-protein conversion factor. The Jones’ (1931) factor has been used extensively, with a standard default conversion factor of 6.25 (Mariotti et al., 2008), which is based on the average nitrogen content and composition of proteins. Data from measured mean values in feces provides a median figure for protein daily loadings of 6.3 g/cap/day with a range of 3.2–16.2 (n = 7) and for nitrogen 1.8 g/cap/day with a range of 0.9–4.9 (n = 18) (Figure 4). Fecal nitrogen is present in the form of undigested dietary protein, nucleic acids, protein from bacteria and shed intestinal mucosal cells as well as being present in secreted mucus (Canfield et al., 1963; Bender and Bender, 1997). Nitrogen can make up 5–7% of the dried solids (Feachem et al., 1978) and of the nitrogen voided in the feces fraction 50% is thought to be water-soluble (Montangero and Belevi, 2007).

Mean endogenous nitrogen excretion in 14 males has been measured at 0.96 g/cap/day in feces, or 38 mg/kg body weight by Calloway and Margen (1971); this is the minimum nitrogen loading that can be expected. The safe rate of nitrogen intake to maintain nitrogen balance is 0.75 g protein/kg body weight/day (FAO/WHO/UNU, 1985) and as a guideline figure of nitrogen voided in feces Bender and Bender (1997) concluded that when a healthy human is in nitrogen equilibrium, nitrogen excretion will equal ±5% of intake. Variation in the protein content of feces is largely dependent on protein intake in the diet; however, the digestion rate of protein has been shown to vary from 69% to 93% as a result of differing types of protein in the diet (Southgate and Durnin, 1970; Calloway and Kretsch, 1978). It should be noted that the majority of nitrogen output is in the urine fraction with this study showing that only 14% is voided through the feces (1.8 g/cap/day) and the majority is excreted in urine (10.7 g/cap/day).

Concentrations of the differing nitrogenous fractions have also been recorded; Silvester et al. (1997) recorded fecal ammonia concentrations on low (68 g/day) and high (192 g/day) protein diets with values of 12 mmol/kg (1.4 mmol/day) and 24 mmol/kg (2.9 mmol/day) respectively. Fecal nitrite levels were also found to be increased twofold on high protein diets, with values of 1678 μg/kg, in comparison to the lower protein diet with 829 μg/kg (Silvester et al., 1997).

#### 3.2.4 Lipids

Fats contribute between 2.4% and 8% of the wet weight of feces (Canfield et al., 1963; Kien et al., 1981; Rivero-Marcotegui et al., 1998; Guyton and Hall, 2000; Wierdsma et al., 2011) or 8.7–16.0% of the dry weight of feces (Calloway and Kretsch, 1978; Tarpila et al., 1978; Stephen et al., 1986). Daily loadings of fat in the fecal fraction from the mean values of 8 studies gave a median value of 4.1 g/cap/day and a range of 1.9–6.4 g/cap/day (Figure 4). However, it should be noted that only one out of the eight studies was from outside Europe and North America (Guatemala): with this individual study presenting the lowest figure in the range of values (1.9 g/cap/day). Age differences have been observed, with infants voiding lower amounts of fecal fat 0.8–3.2 (Shmerling et al., 1970) and children aged 1–11 years voiding 0.9–5.9 (mean 3.0) g/cap/day of fat (Kuo and Huang, 1965). As would be expected fecal fat is positively correlated (*p* <.001) with fecal wet mass and has also been positively correlated with fiber intake (Eastwood et al., 1984). Fecal fat excretion is dependent on dietary intake; however, even with no fat intake excretion of fat occurs. At high levels of fat intake there is no correlation between fat intake and fecal fat excretion (Gades and Stern, 2012). A significant positive correlation (r = 0.56, *p* =.007) between calcium intake and fecal fat excretion was found by Jacobsen et al. (2005) with fecal fat excretion on a high calcium diet increasing from 7% to 18% of dietary fat intake and an increase of 100 mg calcium resulting in an increase of 5.4 g in fat excretion. This increase is thought to be due to an interaction between calcium and fatty acids, which causes insoluble calcium fatty acids to form and resultantly reduces fat absorption and increases fat excretion (Jacobsen et al., 2005). Fat found within feces comes from bacteria and fat in the shredded epithelial cells as well as from the undigested dietary intake of fat (Guyton and Hall, 2000). Broadly the fat content includes substances such as fatty acids, waxes, and phosphoglycerides.

#### 3.2.5 Carbohydrate and Energy Value

The carbohydrate fraction is largely made up of undigested cellulose, vegetable fibers, and pentosan (Canfield et al., 1963). Feces do not contain large quantities of carbohydrates as the majority of what is consumed is absorbed; however, undigested and unabsorbed fractions (RS) remain. A median value (n = 10) of 9 g/cap/day carbohydrate in feces was recorded with a range of 4–24 g/cap/day. The vast majority of studies were again conducted in North America and Europe with only one study in Peru presenting values in the center of this range. The calorific content of feces had a median value (n = 14) of 132 kcal/cap/day (range: 49–347 kcal/cap/day). By using the median value of production (32 g/cap/day) a calorific value of 4115 kcal/kg dry solids can be used as a design standard for calorific value of feces. All studies were carried out in North America and Europe therefore no correlation could be made between income and calorific value. However, the largest quantities of fecal energy are shown from diets containing a large amount of unavailable carbohydrates (Southgate and Durnin, 1970), defined as all polysaccharides not hydrolyzed by the intestinal secretions of humans, as opposed to available carbohydrates such as starch and sugars which result in less fecal energy loss (Southgate, 1973).

#### 3.2.6 Fiber

Human stools contain approximately 25% undigested plant matter, not including any nitrogenous material (Volk and Rummel, 1987). Fiber is present in stools due to the large linked polysaccharides that inhibit digestibility (Volk and Rummel, 1987), therefore the dietary intake will strongly influence the quantity found in feces. The quantity of fiber found in feces (n = 8) ranged from 0.5 to 24.8 g/cap/day with a median value of 6 g/cap/day (Figure 4). Fiber consumption has also been shown to have significant effects on other variables. It was found by Beyer and Flynn (1978) that when a high fiber diet was consumed and compared to a low fiber diet then measurements of fecal fat, protein, carbohydrate, and calories were more than doubled. Similar conclusions were made by Kelsay et al. (1978) when a high fiber diet from fruit was consumed. It was concluded that this was down to fiber consumption having a significant impact on absorption capacity in the gut.

### 3.3 Inorganic Composition

The remaining solids compose the inorganic fraction which is predominantly made up of calcium phosphate and iron phosphate, intestinal secretions, small amounts of dried constituents of digestive juices such as shredded epithelial cells and mucus (Guyton and Hall, 2000; Iyengar et al., 1991). Fixed solids were measured at 3.13 g/cap/day by (Cummings et al., 1996) which was 2.25% of fecal wet weight and 9.02% of fecal dry weight. Fixed solids are in the range of 7.5–16% of TS s (Feachem et al., 1978; Nwaneri et al., 2008; Bai and Wang, 2011); using the assumption of 29 g/cap/day TS then this would give a fixed solid value of between 2 and 4 g/cap/day.

In a healthy fully grown adult the amount of inorganic elements are in equilibrium (Kujawa-Roeleveld and Zeeman, 2006) and are not subject to any transformation within the body (Muñoz et al., 2007). Therefore it would be expected that the intake of elements would be equal to the output in human excreta. The intake of nutrients is therefore of great importance as well as the partitioning of these elements between the two excreta streams of feces and urine. Wignarajah et al. (2003) found that the partitioning of elements between the urine and fecal fractions could be determined by looking at% absorption rates of inorganic elements in the body. Absorption rates were found to be predictable and reliable, therefore if the elemental input of the diet is known for an individual or population (alternatively it could be predicted from recommended daily allowance figures for that population), the partitioning between urine and fecal fractions could be predicted. This is because elements that are absorbed by the body will be excreted in the urine fraction and the remaining fraction will be voided in the feces.

However, absorption rates are not clearly defined at high intake rates; an example cited by Wignarajah et al. (2003) is the partitioning of phosphate. The phosphate absorption rate at normal intake levels is 60%, however, at high rates of phosphate intake the absorption rate is markedly reduced to 40%. This means that at high levels of phosphate intake the relative amount of phosphate voided in feces can be increased from 40% to 60% as the amount absorbed and excreted in urine is reduced.

Minimum and maximum values of elements (Table 5) can be used as an estimate of daily loading rates of elements voided in feces; the variation is likely to be due to the differing dietary intakes which were not recorded. The intake of elements is therefore the most important variable. Therefore, factors that have an effect on this, such as heavy metal contamination of farmland or high concentrations of certain elements, such as lead in the air as a result of industrial pollution, also bear importance. Increased fiber intake has also been shown to lead to an increase in inorganic constituents, particularly Na and P (Southgate et al., 1976). Feachem et al. (1978) recorded% concentration of P, K, and Ca at 3–5.4%, 1–2.5%, and 4.5% respectively in the dried solid fraction. Levels of P in feces have been shown to increase with increasing protein intake; however, protein intake had no other impact on Mg, K, and Ca (Calloway and Margen, 1971). The total quantity of feces voided will also have an impact on the quantity of constituents; Na, K, Mg, Ca, and Zn were all found to be strongly correlated with fecal wet mass (Eastwood et al., 1984).

### 3.4 Chemical Nature

**Figure 5:**
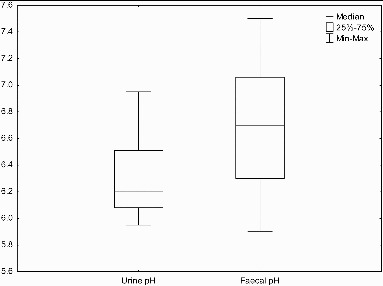
Mean pH values for urine (n = 9) and feces (n = 23).

Fecal pH is neutral with a median value of pH 6.6 and a range of mean pH values of 5.3–7.5 (n = 28) (Figure 5). Fecal pH not only varies between different populations but has also been proven to differ between individuals consuming the same diet and with time (Silvester et al., 1997). Van Dokkum et al. (1983) found a difference of 0.25 in the fecal pH between sampling separated by two days in the same individual when exactly the same diet was consumed.

Fecal pH variation is related to diet (Thornton, 1981; Van Dokkum et al., 1983). Increased dietary fiber was suggested by Newmark and Lupton (1990) to lower fecal pH. However, not all studies have found that high fiber diets correlate with lower fecal pH. In a comparison study of omnivorous and vegetarian diets by Walker and Walker (1992), no significant difference in pH values for the stool or stool water were observed, even though the vegetarian diet provided considerably more fiber. Similarly in a comparative study of omnivorous and vegetarian diets by Van Faassen et al. (1993) no difference between pH values for the stool or the stool water were observed, even though the vegetarian diet again, provided considerably more fiber.

High levels of RS in diets was also shown by Phillips et al. (1995) to lower fecal pH in a controlled experiment of differing RS intakes, a significant inverse relationship between RS intake and fecal pH was found (r = −0.65, *p* <.01). Interestingly 30% of the variance of fecal pH in a study by Van Dokkum et al. (1983) was accounted for by calcium intake, showing a significant positive correlation. Evidence of variation in fecal pH is not conclusive and variation could be due to a specific dietary intake, such as citrus fruit which has been proven to lower fecal pH (Walker et al., 1979).

### 3.5 Physical Form

For the development of onsite treatment technologies an understanding of the physical form of feces is important; this characterization can be done through the use of visual scales or prevalence rates of diarrhea and constipation.

#### 3.5.1 Visual Scale

**Figure 6:**
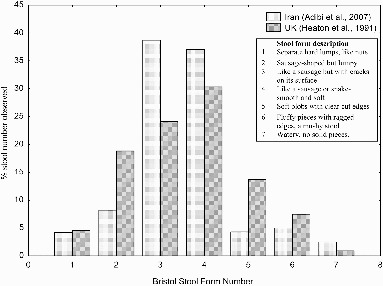
Data from two separate studies of healthy subjects (Heaton et al., 1992; Adibi et al., 2007) both use the Bristol Stool Form scale. Stool types 3 and 4 make up the most common stool type in both studies, however all types of stool are recorded in both studies.

Within the medical literature a number of linear scales have been used to characterize feces e.g., Davies et al. (1986), however, with different scales in use cross comparison of studies is difficult. The most popular scale used is that of Lewis and Heaton (1997) who proposed the “Bristol Scale Stool Form” (Figure 6). This simplified visual scale provides an indication of the form of feces expected and the variation that can be observed across a population. Stool form is considered abnormal when type 1, 6, and 7 occurs and this is 15% of the time within a healthy population (Heaton et al., 1992). The mean value for a general population sample of 66 people using the Bristol Stool Form scale have been placed at 3.6 by Lewis and Heaton (1997). The distribution of the physical form in two populations of differing countries shows that stool types 3 and 4 are most commonly reported (Figure 6). Variation occurs between individuals, by age and gender (Heaton et al., 1992), although diet and health prove more important variables (Davies et al., 1986; Heaton et al., 1992). Dietary fiber is linked to stool texture, as dietary fiber increases stools become softer (Davies et al., 1986).

#### 3.5.2 Diarrhea

Diarrhea has an impact on stool production, structure, form, and composition.

In a controlled study by Wierdsma et al. (2011) it was found that patients in an intensive care unit with diarrhea had over five times the wet fecal weight (796 g/cap/day versus 157 g/cap/day) compared to those without diarrhea. Increased water losses are the predominant cause of the increase in weight; an increase in water content of 5% was shown by Wierdsma et al. (2011) and in a study by Goy et al. (1976) feces of patients with diarrhea had a significantly (*p* <.05) greater percentage water content compared to control subjects. fecal water loss of more than 10 mL/kg body weight is often used as a definition of chronic diarrhea (Auth et al., 2012). Those with diarrhea display higher fecal protein losses of 16.2 g/cap/day versus 5.6 g/cap/day and higher fecal energy losses were also shown in comparison to patients with normal stools (Wierdsma et al., 2011). However, fecal energetic content per gram of feces (kcal/g wet feces) was not significantly different between subjects with and without diarrhea (Wierdsma et al., 2011).

Diarrhea is defined as a minimum of 3 liquid stools per day; it is further sub-divided into acute diarrhea (defined as diarrhea lasting up to 3 weeks) and chronic diarrhea (lasting any longer than 3 weeks) (Patel and Thillainayagam, 2009). It has been classified as stool types 6 and 7 on the Bristol Stool Form Scale (Figure 6). Chronic diarrhea prevalence rates in five studies across the UK, US, and Asia show an average of 4.6% (Table 7) with prevalence more frequent[Table T0008]in the elderly at rates of 14.2% (Talley et al., 1992). Acute (infectious) diarrhea is caused most commonly by viruses, bacteria, and protozoa and is commonly transmitted by the fecal-oral route through water, food, and person to person contact (Farthing and Kelly, 2007). Acute diarrhea prevalence figures have been applied to geographic areas, such as in the United States where there is an equivalent of 1.4 episodes per person per year (Herikstad et al., 2002) and in the UK with just under 1 episode per person per year (Feldman and Banatvala, 1994).

**Table 7:** Diarrhea prevalence in a selection of six countries

Study	Country	n	Chronic diarrhea prevalence (%)
Han et al. (2006)	Korea	1066	6.6
Chen et al. (2000)	Singapore	271	7
Danivat et al. (1988)	Thailand	1077	2.3
Danivat et al. (1988)	UK	301	4.7
Sandler and Drossman (1987)	UK	1128	3.6
Danivat et al. (1988)	USA	789	4.9
Tan et al. (2003)	Malaysia	84	3
Average across studies		7	4.6

**Table 8 T0008:** Per Capita Generation of Components in Urine

Variable	Range (median) (g/cap/day)	References
Total N (n = 8)	2–35 (11)	
Urea	10.00–35.00	Bender and Bender (1997)
	1.36–6.77	Calloway and Margen (1971)
Ammonia	0.34–1.2	Bender and Bender (1997)
Creatine	0–0.15	Dong (1999)
	<0.10	Bender and Bender (1997)
Creatinine	0.001–0.002	Bender and Bender (1997)
	1.640	Dong (1999)
	1–1.800	Harper et al. (1977)
Uric acid	0.25–0.75	Bender and Bender (1997)
	0.86	Dong (1999)
	0.50–0.80	Harper et al. (1977)
Total P	0.93	Jönsson et al. (2005)
	0.62–0.74	Taylor and Curhan (2006)
	0.45–0.71	Borawski et al. (2008)
	1.15–1.30	Calloway and Margen (1971)
Total K	0.78–2.50	Wignarajah et al. (2003)
	2.5	Del Porto and Steinfeld (1999)
	0.027–0.036	Borawski et al. (2008)
	2.51–2.87	Calloway and Margen (1971)
Na	3.45–4.53	Wignarajah et al. (2003)
	0.082–0.163	Borawski et al. (2008)
SO_2_−4	1.34–1.63	Taylor and Curhan (2006)
Ca	0.20–0.50	Wignarajah et al. (2003)
	0.118–0.113	Taylor and Curhan (2006)
	0.057–0.134	Borawski et al. (2008)
	0.14–0.25	Calloway and Margen (1971)
Mg	0.19–0.21	Calloway and Margen (1971)

Acute diarrhea prevalence is higher in low income countries as many of the risk factors of contracting diarrheal illness are associated with poor socioeconomic conditions (Ahs et al., 2010). Factors that increase exposure to infectious diarrhea include lack of access to safe water supplies, inadequate sanitation facilities, and poor personal hygiene. Added to this factors that reduce resistance to infection are also important such as age, malnutrition, and illnesses such as the human immuno-deficiency virus (HIV) (Ahs et al., 2010). Geographically, there is an overlap of areas with a large burden of diarrheal illness and those with a large proportion of HIV cases; some enteric pathogens have also been shown to occur more frequently in HIV-positive individuals than in the general population, including campylobacter, cryptosporidium, and shigella (Ahs et al., 2010). Zinc and vitamin A deficiencies have also been shown to increase susceptibility to diarrhea episodes, especially in children (Walker and Black, 2004).

Diarrhea disproportionately affects children in low and middle income countries due to inadequate water and sanitation facilities and nutritional risk factors (Fischer Walker et al., 2012). In a systematic review by Fischer Walker et al. (2012) diarrhea prevalence rates in children were estimated at 2.9 episodes/child year, with incidence rates the highest among infants aged 6–11 months. In an overview report by the World Bank, data collected by a Demographic and Health Survey (DHS) project between 1990 and 2005 was presented by Gwatkin et al. (2007) with prevalence measured according to the% of children under 5 who had diarrhea in the 2 weeks prior to the survey; population averages for the regions of South Asia (15.3%), Sub-Saharan Africa (19.7%), East Asia, and the Pacific (13%) were recorded (Gwatkin et al., 2007). Infectious diarrhea is also more common among elderly populations due to increased incidence of immunodeficiency and resultantly an increased likelihood of bacteria in the blood (DuPont, 1997).

Seasonality affects the prevalence rates of diarrhea. It has been observed that acute diarrhea becomes an epidemic in the rainy season in places such as Kathmandu (Karki and Tiwari, 2007) this is largely due to the problem of water supply contamination. However, in a cross-sectional study of diarrhea in children under 5, a negative association between rainfall and diarrhea rates was found by Lloyd et al. (2007) with a 4% increase in diarrhea incidence (95% confidence interval, CI: 1–7%, *p* =.02) for each 10 mm month^−1^ decrease in rainfall, this was thought to be due to the use of unprotected water sources during water scarcity.

#### 3.5.3 Constipation

Constipation has prevalence rates that can range from 1.9% to 27.2% in an American population (Higgins and Johanson, 2004); however, it is commonly found at 6–12% in a general population (Heaton et al., 1992; Talley et al., 1993; Thompson et al., 2000). Constipation increases with increasing age, particularly after the age of 65 (Higgins and Johanson, 2004). Only one comparative study (Aichbichler et al., 1998) of fecal characteristics of constipated and non-constipated subjects was found; concluding that stool weight per week was markedly reduced in constipated subjects due to a reduction in stool water and TS output. There are numerous other studies that report fecal weights of constipated subjects, e.g., (Ashraf et al., 1996; Chen et al., 2008) these studies report daily per capita weights that fall within the study range presented (for example, in a study of constipated subjects by Chen et al. (2008) values of 108.3 g/cap/day were recorded, in comparison to the median value of 128 g/cap/day reported in this study); however, shorter experimental studies can often be misleading and it is often the case that over prolonged study periods of weeks or even months stool weights can be considerably decreased (Aichbichler et al., 1998).

### 3.6 Urine

In contrast to feces, the characteristics of urine have been studied extensively (Diem and Lentner, 1970; Kirchmann and Pettersson, 1994; Karak and Bhattacharyya, 2011). Urine as a potential fertilizer has attracted much attention in the treatability sector with a large range of literature exploring the agricultural fertilizer potential (Palmquist and Jönsson, 2004; Karak and Bhattacharyya, 2011; AdeOluwa and Cofie, 2012). Urine presents less danger to human health in comparison to feces and contains few enteric microorganisms, however, some human pathogen microorganisms such as *Schistosoma haematobium*, *Salmonella typhi*, *Salmonella paratyphi*, and *Leptospira interrogans* as well as helminth eggs can be found in the urine fraction (Feachem et al., 1978; Heinonen-Tanski and van Wijk-Sijbesma, 2005).

#### 3.6.1 Liquid Generation

Human urine is a liquid that is secreted by the kidneys, collected within the bladder and excreted through the urethra. Urine is composed of 91–96% water (Drangert, 1998; Höglund et al., 2000; Heinonen-Tanski et al., 2007) and the remainder can be broadly characterized into inorganic salts, urea, organic compounds, and organic ammonium salts (Putnam, 1971).

Liquid generation from humans is dependent on the water balance of individuals. Liquid output is in the form of urine, fecal water, from the skin through sweating, and from the lungs through respiration. A median volume of 1.4 L/cap/day urine is excreted with mean values ranging from 0.6 to 2.6 L/cap/day (n = 14). In medicine, urine output is used to assess circulatory adequacy with inadequate urine output considered at <0.5 mL/kg body weight/hour for adults (Suen et al., 1998) and at 1–1.5 mL/kg body weight/hour in children (Yowler and Fratianne, 2000). This indicates the minimal urine output that can be expected.

Variation in total urine output (Figure 3) is primarily due to fluid intake and in a study by Parker and Gallagher (1992) accounted for 78% of the variation observed in a sample of 11,748 days’ worth of data. It was noted by Garrow et al. (1993) that the volume of water drunk as fluid is generally equal to the volume of urine produced. Body size is inevitably important when assessing a human's urinary output; when assigning loading rates in wastewater, Almeida et al. (1999) reduced urinary output by 33% for children such that Karak and Bhattacharyya (2011) stated that children urinate about half that of the volume excreted by adults. Urine output therefore increases with body size. Other factors leading to variation such as excessive exercising or sweating will have an effect on the quantity of urine generated as they will impact hydration. Variation in urine output according to race has been proven significant with the urine volume of black women 0.24 L/day less than white women (*p* =.001) (Taylor and Curhan, 2007). It was also observed by Clark et al. (2011) that higher volumes of urine tended to be from subjects who were older, were more likely to be obese or taking medication.

Information regarding the number of times urination takes place over a 24 hr period is sparse and is likely to vary greatly due to fluid intake, biological factors, and health of the individual. Schouw et al. (2002) recorded a figure of 5.4 urinations per day in a boy's prison in Thailand and Bael et al. (2007) reported a median figure of 6 urinations/24 hr (range of 2–11 urinations/24 hr) in a study of children aged 6–12 years. A figure of 8 urinations per 24 hr period was recorded for a population sample in the United States (n = 17) (Clare et al., 2009). The diurnal variation of urinary output is not commonly recorded, however, a control sample of 15 healthy adult subjects showed that 60% of total urine volume was excreted during the daytime (09:00–21:00) and 40% was excreted at night time (21:00–09:00) (Hineno et al., 1994).

#### 3.6.2 Composition

Urine composition varies due to differences in physical exercise, environmental conditions, as well as water, salt, and high protein intakes. Urine osmolarity is a measure of the water distribution amongst fluid components. It can vary between 50 and 1200 mOsmol/kg, with the average urinary excretion of solute 1000 mOsmol/cap/day (Garrow et al., 1993; Callis et al., 1999). This solute is excreted in a median volume of 1.4 L/cap/day of urine. The quantity of solute varies between individuals and with differing diets; for example, the high consumption of meat leads to larger volumes of solutes as meat is a major source of urea (the largest solute fraction) as well as potassium and phosphates, whereas vegetarian diets are likely to lead to reduced solute production as most energy is derived from carbohydrate (Garrow et al., 1993).

The median value of mean total urine solids loading rates is 59 g/cap/day (n = 7) and mean values range from 57 to 64 g/cap/day. The dry matter of urine was measured at 4.7–10.4 g/L by Heinonen-Tanski and van Wijk-Sijbesma (2005). The concentration of total suspended solids has been recorded at 21 mg/L (Almeida et al., 1999) and total dissolved solids have been recorded at 31.4 mg/g (Putnam, 1971). Organic matter makes up between 65% and 85% of urine dry solids (Strauss, 1985), with volatile solids comprising 75–85% of TS (Fry and Merrill, 1973; House, 1981). Urea is the most predominant constituent making up over 50% of total organic solids, and is produced through the metabolism of protein. The other major solutes excreted in urine are Na and K, which are largely derived from dietary intake.

#### 3.6.3 Chemical Composition

Dry urine solids are composed of 14–18% N, 13% C, 3.7% P, and 3.7% K (Strauss, 1985). Concentrations of major elements in urine were recorded at 6.87 g/L carbon, 8.12 g/L nitrogen, 8.25 g/L oxygen, and 1.51 g/L hydrogen by Putnam (1971). Of the feces and urine fractions, urine contains the largest proportion of N (90%), P (50–65%), and K (50–80%) released from the body (Heinonen-Tanski and van Wijk-Sijbesma, 2005).

Nitrogen is predominantly in the form of organic nitrogen and mostly in the form of urea (Beler-Baykal et al., 2011). Median values of total N excretion of 11 g/cap/day were recorded (n = 8) with a range of mean values from 2 to 35 g/cap/day. Endogenous total N excretion of 13 men with the absence of protein in the diet was 2.41 g/cap/day, with no correlation with body weight found (r = 0.450) (Calloway and Margen, 1971). This therefore provides a minimum figure for N excretion. The dietary intake of protein is the most predominant factor effecting N excretion. Urinary N components increase with increasing levels of protein in the diet; a positive correlation (r^2^) between urinary N and protein intake (intake ranging from 51 to 212 g/day) was found to be 0.91 (Magee et al., 2004). In a meta-analysis of data by Kipnis et al. (2001) it was found that urinary N is 80% of dietary intake on average.

Of the nitrogenous fractions urea is the most predominant, making up between 75% and 90% (Lentner, 1981). Urea concentrations range from 9.3 to 23.3 g/L (Putnam, 1971; Otterpohl et al., 2002; Jönsson, 2005), with daily loadings of 1.4–35.0 g/cap/day (Calloway and Margen, 1971; Bender and Bender, 1997). Creatinine is a significant nitrogenous fraction in urine. Endogenous creatinine was measured at 1.59 g/cap/day and was correlated with body weight (22 ± 4 mg/kg, r = 0.918) and is also dependent on age and muscle mass (Calloway and Margen, 1971). Concentrations can vary according to gender with male subjects recording higher (*p* =.001) creatinine values than female subjects, 1.9 and 1.4 respectively (Newman et al., 2000). Concentrations of creatinine in urine also decreases when increasing volumes of urine are excreted over a 24 hr period (R^2^ = 0.618, r = 0.786, *p* <.001) (Newman et al., 2000). If there has been incomplete sampling over 24 hr an internal standard against the creatinine value can be used, with standards of creatinine excretion set at 1.7 g/day in men and 1.0 g/day in women (Jackson, 1966). Nitrate concentrations in urine are low, with measured values at 1.07 mmol/L and 2.06 mmol/day when a high protein diet is consumed (192 g/day) and 1.09 mmol/L and 2.23 mmol/day when a lower protein diet is consumed (68 g/day) (Silvester et al., 1997).

Protein intake is the predominant cause for variation in nitrogen concentrations of urine. In addition to this, protein intake has also been shown to impact other mineral constituents in urine. For example, in very low protein diets P and K were shown to be increased, Ca was reduced in very low protein diets but protein intake had no effect on Mg concentrations in urine (Calloway and Margen, 1971).

Differences in chemical composition have been observed according to race by Taylor and Curhan (2007) with black women (n = 146) excreting 65 mg less Ca (*p* <.001), 351 mg less K (*p* <.001), 11 mg less Mg (*p* <.001), and 120 mg less P (*p* <.001) per day than white women (n = 330); these observations were consistent even after adjustment for age and body mass index (BMI). Animal protein in the diet has been shown to lead to increased levels of urinary calcium, with calcium excretion at 21% of intake whereas with higher levels of vegetable protein calcium excretion is 16% of intake (Taylor and Curhan, 2007). Positive associations were found between BMI and urinary calcium excretion, however, it was concluded that this was due to differences in animal protein and sodium intake (Taylor and Curhan, 2006).

#### 3.6.4 Chemical Nature

The pH of fresh urine is largely neutral with a median of pH 6.2, with a range of mean pH values of 5.5–7.0 based on a large subject sample size across nine individual studies (Figure 5). There are numerous factors that can lead to changes in urinary pH but diet once again provides a key variable. Urinary pH is reduced by high protein intake through meat and dairy produce as well as through alcohol consumption (Kanbara et al., 2012). However urine is more alkaline with the ingestion of potassium and organic acids which are increased in diets with high consumption of vegetables and fruit. Taylor and Curhan (2007) found that black women had a higher urinary pH than white women by 0.11 units (*p* =.03) even when adjusted for differences in diet, BMI, and age. Further, an inverse relationship between BMI and urine pH (*p* =.02) was found by Taylor and Curhan (2006). Factors leading to a lower urinary pH include a higher weight, old age, and increased dietary acid intake (Hesse et al., 1986; Maalouf et al., 2004; Taylor and Curhan, 2007).

The specific gravity of urine ranged from 1.002 to 1.037 in spot samples of 534 subjects (aged 18–68) with a high correlation (r = 0.82, *p* <.001) observed between creatinine and specific gravity (Carrieri et al., 2000). The COD levels of 8–17 g/L found in urine are low (Table 9); this is likely to be because most of the organics excreted are small molecules. The mean calorific content of urine was measured at 100 kcal/day (range: 91–117) by Southgate and Durnin (1970): using the median value of urine solids produced daily (59.0 g/cap/day) a design value of 1707 kcal/kg can be used.

**Table 9:** Concentration of key components in fresh urine

Variable	Concentration range (mg/L)	References
Electrical conductivity EC	160 mS/cm	Jana et al. (2012)
	270 mS/cm	Jönsson et al. (1997)
Osmolarity	1025 mosmol/kg	Callis et al. (1999)
	50–1200 mosmol/kg	Garrow et al. (1993)
COD	17,500	Putnam (1971), Almeida et al. (1999)
	6270–10,600	Putnam (1971)
Total N	8000	Ban and Dave (2004)
	5000	Jönsson et al. (2004)
	11,000–13,900	Jönsson et al. (2004), Southgate and Durnin (1970)
	4000	Jönsson et al. (1997)
	12,000	Mojtahedi et al. (2002)
	11,700	Beler-Baykal et al. (2004)
TKN	9220	Beler-Baykal et al. (2011)
	5580–7900	Putnam (1971)
Urea	21,400	Jönsson (2005)
	9300–23,300	Putnam (1971)
	10,000	Otterpohl et al. (2002)
NH_4_-N	125	Jana et al. (2012)
	600	Beler-Baykal et al. (2004)
NH_3_-N	480	Tilley et al. (2008b), Diem and Lentner (1970)
	200–730	Putnam (1971)
	300	Tilley et al. (2008a)
Total P	350	Jönsson et al. (1997)
	800–2500	Wignarajah et al. (2003)
	1000	Del Porto and Steinfeld (1999)
	1800	Ban and Dave (2004)
PO_4_-P	205	Tilley et al. (2008a), Diem and Lentner (1970), Jana et al. (2012)
	450	Tilley et al. (2008a)
	760	Diem and Lentner (1970)
K	966–1446	Beler-Baykal et al. (2004)
	1200	Jönsson et al. (1997)
	750–2610	Putnam (1971)
Ca	230	Diem and Lentner (1970)
	32	Jana et al. (2012)
	70	Tilley et al. (2008a)
Mg	120	Diem and Lentner (1970)
	70	Tilley et al. (2008a)
Creatine	0–890	Putnam (1971)
Creatinine	311–2150	Putnam (1971)
Uric acid	40	Putnam (1971)
	152–858	Jen et al. (2002)
	856	Dong (1999)

### 3.7 Additional Influences on Treatment Systems

Both fecal solids (29 g/cap/day) and urine solids (58–64 g/cap/day) are produced daily in large quantities. A mixed stream treatment system at source will therefore have to deal with a large quantity of solids from both feces and urine. However, it is also the case that feces and urine are likely not to be the only additions to a treatment system. A treatment system may also have to deal with additional material from human behavioral practices such as the use of toilet paper or the addition of sanitary items (Table 10). A similar principle applies to water addition; a large liquid fraction is produced daily through urine and fecal output; however this may be further increased by additional water inputs such as pour flush toilet systems or anal cleansing practices.[Table T0011]

**Table 10:** Components and generation rate of human excreta waste streams and possible additional inputs

Component of solids fraction	Generation rate (g/cap/day)	Component of liquid fraction (L/cap/day)	Generation rate
Stool mean (range) g/cap/day	32 (4–102)	Stool water mean (range)	0.101 (0.053–0.265)
Urine	61 (50–75)	Urine median (range)	1.42(0.8–2.45)
Toilet paper use average	11.68–19.4^bc^	Anal cleansing L/wash	0.35–3^de^
Toilet paper use men	6–10.3^abc^	Pour flush toilet water L/flush	1–3^f^
Toilet paper use women	17.9–36^abc^		
Menstrual pads and flow	34^a^		
Sanitary Items. refuse item/cap/day	0.16^b^		

^a^Parker and Gallagher (1992), ^b^Friedler et al. (1996), ^c^Almeida et al. (1999), ^d^Strauss (1985), ^e^Tilley et al. (2008b), ^f^Cairncross and Feachem (1993).

**Table 11 T0011:** Classifications of broad treatment pathways in wastewater treatment

Process type	Examples	Resource recovery
Biological	Anaerobic digestion	Biogas
	Decoupled HRT and SRT	Digestate/Biosolids/liquid fraction
	UASB	Biofuel production
	Wet and dry composting	Compost fertilizer
Thermal processes	Pyrolysis/gasification	Energy/Char
	Incineration	Energy/Ash
Separation	Biofiltration	Pathogen free water
	Membrane pervaporation	Pyrolysis
Chemical processes	Electrochemical disinfection	Pathogen free products
	Ammonia disinfection	NPK irrigation water/fertilizer
	Struvite	Phosphorus
	Ammonia stripping	Fertilizer

## 4. DISCUSSION

Existing OSS facilities are often poorly designed, constructed, and maintained which regularly results in inadequate sanitation facilities in many low income regions. This problem has given rise to research into the on-site treatment and/or resource recovery from feces and urine within a low income context. This trend has accelerated with the challenge presented to researchers by the Bill and Melinda Gates foundation to “Reinvent the Toilet” (Global Development Program, 2013). A large proportion of this research aims to treat feces and urine as a fresh waste stream on the site of production, giving a need to understand the production, composition, and any variation around these factors in order to determine how this may impact these technologies. In this discussion all types of conventional treatment processes were considered alongside recent research funded by the Bill and Melinda Gates Foundation (BMGF). These grants (Sustainable Sanitation Alliance, 2013) were grouped according to their treatment pathways comprising; biological processes (17), physical separators (7), chemical processes (3), and thermal processes (8). The principle aim of this discussion is to understand how the production rates, physical and chemical composition of feces and urine can lead to an improved understanding of potential treatment pathways that are either currently in use or under development in the OSS technology sector.

### 4.1 Biological Processes

The predominant factors likely to impact biological processes to the greatest extent are solids loading, energy content, protein, and fat concentration in the feces and the high urea concentrations in urine.

The high solids loading rate associated with fresh feces (∼25% wt.) when viewed as an individual waste stream presents a potential barrier to the successful implementation of high rate anaerobic systems in relation to their solids handling and rheological impacts on mixing and pumping (Speece, 2008). Accordingly, high solids anaerobic digestion processes (operating with solids concentrations greater than 15% w/w) represent a more appropriate match due to the significantly lower impact associated with mixing. Operation at the higher solids loadings will translate to smaller reactor volumes, lower energy requirements, and less material handling than traditionally encountered with standard anaerobic digestion (Guendouz et al., 2008) but would most likely result in a reduced rate and lower biogas yields. For biological processes such as aerobic composting the optimum moisture content is 30–60% (Liang et al., 2003): the moisture content of feces was greater than this (75%) increasing the potential for anaerobic conditions to develop due to water logging (Tiquia et al., 1996). Therefore, incorporation of dewatering pretreatment or a cocomposting feedstock should be considered in order to establish resilient conditions to maximize the efficacy of the desired aerobic degradation pathways. Importantly, the fluctuating levels of moisture content reported in feces (63–86%) means that amendment strategies need to be appropriately flexible and robust and are likely to require a degree of bespoke commissioning.

Based on the COD values collected in this study each 66 g/cap/day COD added and removed by a digester could theoretically produce 0.0175 m^3^ of methane at standard temperatures and pressures (Grady et al., 1999). Practical delivery of such potential is dependent on anaerobic reactor type, retention time, and biodegradability such that actual conversion of the available organic matter to biogas is expected to range between 40% and 90% (Mang and Li, 2010). For instance, a key variable is associated with the fiber content of feces which was found to vary widely (Figure 4); especially in populations consuming high fiber diets (such as diets consumed in low income countries). The importance of this relates to the relatively lower biodegradation rate of the fibrous material resulting in reduced COD conversions. Importantly, increased wet mass production rates above the average (128 g/cap/day) are commonly associated with increased levels of indigestible fiber in the feces. Accordingly there is a poor correlation between wet mass loading and energy production. Whilst this places a risk of overestimation during design for such systems the impact can be readily accounted for as the fiber content of feces is directly dependent on the non-degradable fiber intake of the population within the associated catchment. Consequently, the fiber composition of feces for a given population can be predicted if diet is known and accounted for in such calculations.

Potential biogas production from feces could therefore be significant, however, the relatively small quantities of solids produced per cap/day should be noted and may mean that in order for significant quantities of methane to be produced a large population would be required or an additional codigestion feedstock. This factor is likely to be problematic to small household or community anaerobic digester designs that cite methane production as a key driver for gaining energy neutral systems or for additional cost recovery.

The efficacy of biological processes for the treatment of feces and urine, in either aerobic or anaerobic processes, may be inhibited through imbalances in the macronutrient composition of such streams. For instance, anaerobic digestion proceeds optimally when the C:N ratio is around 20:1 to 30:1 (Parkin and Owen, 1986); this is not the case in feces (8:1), urine (0.8:1) or as a combined waste stream (2.3:1). Similarly, in aerobic systems the recommended ratio for C:N:P (100:10:1 to 100:5:1) (Tchobanoglous et al., 2003) would not be reached. However, imbalances in the macronutrient composition could be rectified through the use of organic waste substrates that are frequently locally available and could be a simple means of increasing the viability of biological systems.

Potential chronic toxicity for treatment by anaerobic processes can be assessed according to the moderately inhibitory and strongly inhibitory concentration classifications according to Parkin and Owen (1986). Feces as a single waste stream showed concentrations of Na^+^, K^+^, Ca^2+^, and Mg^2+^ that were of moderately inhibitory concentrations with values of K^+^ reaching levels defined as strongly inhibitory on occasions. Toxic metals such as Cu, Ni, Cr, and Pb were not of significant concentrations to inhibit anaerobic processes of a feces waste stream. However, the high concentrations of sulfide reported have the potential to exhibit toxicity to methanogenic bacteria (Speece, 2008); this will only occur when high levels of sulfate are entering digesters along with sulfate reducing bacteria. Relatively high levels of sulfate 1.34–1.63 g/cap/day were recorded in urine but with very small amounts of elemental sulfur found (0.16 g/cap/day) in the feces fraction.

Nitrogen excreted in urine and voided in feces was shown to vary according to diet (primarily levels of protein intake) and combined median daily losses (13 g/cap/day) could have the potential to lead to ammonia toxicity problems. Ammonia (NH_3_-N) concentration is a function of ammonium (NH_4_^+^) concentration, temperature and pH (Speece, 1996); thresholds in anaerobic systems can be found at concentrations of 100–500 mg/L depending on adjustment time (Tchobanoglous, 2003). Measurements of ammonia in feces are within this range (204–409 mg/kg), although a significant proportion of protein (29 g/kg) was found in the feces fraction that will degrade to produce additional ammonia, dependent on storage time and conditions. The addition of urine to this waste stream (urine comprises 80% of total N losses) could lead to ammonia threshold limits being exceeded in an undiluted waste stream. This is because a large proportion (>80%) of the nitrogenous fraction of urine is in the form of urea, which in turn breaks down into ammonia. Therefore, ammonia toxicity (resulting from urea toxicity) is likely to be problematic when feces and urine are treated as a combined waste stream and significant dilution could be necessary. Toxicity from the urine fraction could have negative impacts on biological systems as relatively large volumes of urine are collected in relation to feces (daily urine:feces ratio on a weight basis of 11:1). Accordingly it is suggested that smaller household systems that treat a combined feces and urine waste stream need to especially consider such issues and may be enhanced through inclusion of source separation. Source separation could be carried out through the use of urine diverting toilets in which the feces and urine fractions are collected separately within the toilet bowl.

### 4.2 Physical Separators

There are numerous different types of separating technologies; however, the majority are likely to be predominantly influenced by variation in the solids content, physical form, as well as levels of protein and fat in feces.

For technologies based on separation the lack of a standard feces shape, structure, and water content may be one of the greatest challenges. This could impact bound water removal from different stool types and also the different particle sizes that make up feces. This uncertainty could be problematic when selecting process types and optimization operating conditions. In addition to this feces show a low proportion of fixed to volatile solids which could make dewatering challenging and require the addition of increasing amounts of chemicals or conditioning agents in order to gain adequate separation without pretreatment.

Significant levels of protein in the feces fraction (29 g/kg) and the potential for fluctuations in this value (range of 19 to 122 g/kg) may be unfavorable to separation processes such as membrane and other surface filter systems. Layers of protein that form on the outside of particles could lead to clogging and its deposition and adhesion to membrane surfaces may cause fouling (Chan and Chen, 2004). Similarly fat can be problematic to separation technologies as it can act as a binder for particles (Nguyen et al., 2012). Fat content in feces shows variation across studies (Figure 4) but remains within a narrow region (5.8 to 49.1 g/kg). The concentration of fat in feces (median of 25 g/kg) is comparatively low in comparison to conventional types of wastewater sludge such as primary sludge which has much higher levels of fats, oils and greases: this is usually due to the discharge of these products in the sewage system. Nevertheless, shock loads due to variation in the fat content of feces may be large enough to cause the clogging of pores and impact dewatering properties.

Information regarding the physical structure immediately after voiding provides an indication as to how the structure of feces may change over short time periods, for example, in the Bristol Stool Form scale a number of 1 or 2 would suggest a feces structure that holds its shape to a much greater extent than others in the scale. Studies were found regarding the settling and thickening of excreta from septage and public toilet tanks (Heinss et al., 1999) but in this review no studies were found regarding the change in the physical structure of feces once voided over shorter time scales. This lack of data regarding the change in physical structure over time is limiting current ability to fully understand technology needs. Importantly, the time required to lose the initial consolidated identity of the fresh fecal material is required to understand the potential virtue of utilizing fast separation processes that could benefit from the initial cohesion of the solid material. However, such development must also take into account looser fecal material that will also enter such systems and is likely to be significantly less effectively removed by physical processes. Accordingly, understanding the kinetics of the structural change in fecal material during the initial periods after generation remains a critical area for future research activity that could inform novel low cost technology development.

### 4.3 Chemical Processes

Chemical treatment processes can be wide ranging and are dependent on the end use and initial purpose of treatment and include processes such as chemical precipitation, disinfection, oxidation, neutralization, and stabilization.

Perhaps the most obvious process relates to precipitation of the available phosphorus, magnesium, calcium, and sulfur along with the other micronutrients that exist within fecal material and urine (Table 9), in particular the use of source separation to enable recovery of the high content of P in urine (0.4–2.5 g/cap/day) through struvite precipitation. The pH of feces and urine are both slightly acidic in nature (Figure 5), however, the pH level is likely to increase over short time periods which helps drive the precipitation reactions. Indeed, this self-induced onset of precipitation can be detrimental to treatment technology through the precipitation of unwanted scale forming crystals and is considered a particular problem in the supernatant following solid/liquid separation. Nevertheless, the nutrient potential of feces should not be underestimated, with 50% of N being water-soluble as well as 40% of total P excretion being voided in the feces.

### 4.4 Thermal Processes

Efficient thermal technologies have been the focus of much development because of their potential for energy saving and cost recovery. However, although there is great potential for energy production there is the negative aspect of the loss of nutrients present within feces and urine as the majority are made unavailable for agriculture use. The cost efficiency of the process is primarily dependent on the water content of excreta and its calorific value.

The TS content of feces and urine is likely to be the most important factor impacting thermal treatment technology, with TS content of feces (25%) and urine (1%). The TS content and its variation will determine the financial viability of thermal processes and whether it can be a viable feedstock. However, the TS content of feces (25% TS), is in a similar range to that of dewatered sludge (typically 22–36% TS) from conventional sewage treatment works using belt-filter press, filter press, and centrifuge dewatering (Tchobanoglous et al., 2003). This is important as it highlights that when feces are voided the material is already at the level of de-watered sludge if it could avoid being diluted. This could therefore mean that thermal treatment technologies could potentially be used without prior dewatering processes and this factor could promote collection practices that involve less dilution of the waste stream highlighting again the need to understand the time related change in fecal identity that occurs during the initial periods after being voided.

Variation in water content (Figure 3) was significant with a range of 63–86%. Diet was the predominant cause for variation in water content (predominantly fiber intake) in healthy subjects, however, in unhealthy subjects this range can further increase due to the prevalence of diarrhea. Chronic and acute diarrhea within populations could have a significant impact on treatment technology as feces of those with diarrhea showed increases in water content and a change in physical structure. Global averages of diarrhea prevalence are significant in developed countries; therefore, this should be accounted for and amplified for technologies aimed at low income regions where both the chronic and acute diarrhea prevalence rates are likely to be significantly greater. In contrast to diarrhea, constipation decreases the water content of feces and is equally prevalent in the developed world. Scales relating to the physical form of feces also provides a further estimation of the solids composition by providing approximate estimations of the TS content of feces across large sectors of populations. Research being carried out by Wooley et al. (2013) into assigning a TS value to the Bristol Stool Form scale will be of further benefit to technology development in this respect. Extremes in solids composition may cancel each other out in an averaging effect; however, thermal systems would have to be capable of dealing with this wide range and potential fluctuations in water content.[Table T0012]

**Table 12 T0012:** Summary table of feces and urine characteristics providing on-site sanitation design criteria

Key design criteria	Median value
*Feces*	
Fecal wet weight (g/cap/day)	128
Fecal dry weight (g/cap/day)	29
Stool frequency (motions/24 hr)	1.1
Total solids (%)	25
VS (% of TS)	89
COD (g/cap/day)	71
Nitrogen (g/cap/day)	1.8
Protein (g/cap/day)	6.3
Lipids (g/cap/day)	4.1
Carbohydrate (g/cap/day)	9
Fiber (g/cap/day)	6
Calorific value (kcal/cap/day)	132
pH	6.6
*Urine*	
Urine wet weight (L/cap/day)	1.4
Urine dry weight (g/cap/day)	59
Urination frequency (urinations/24 hr)	6
Nitrogen (g/cap/day)	11
Calorific value (kcal/cap/day)	1701
pH	6.2

The calorific value can be used as a metric of potential energy that can be produced during combustion of excreta. Calorific value of feces (4115 kcal/kg) shows lower values in comparison to animal manure feed-stocks such as swine (4634 kcal/kg), similar values to cattle manure (4211 kcal/kg) but greater than poultry litter (3611 kcal/kg) (Cantrell et al., 2012). Human feces therefore could present an economically viable option for energy creation through combustion. However, humans will consume a much more varied diet then animals, leading to greater deviation from median values than would be seen in manure feedstock. For example, although there is variation in the calorific value of swine manure from different sites (e.g., 4660–7887 kcal/kg (Cao et al., 2010; Xiu et al., 2010) variation within these sites is limited as the animals are kept under the same conditions and are being fed the same diet. In contrast, variation in the energy value of feces is quite substantial (1523–10,875 kcal/kg). This variation is predominantly caused by the varying presence of unavailable carbohydrates in the diet, the larger the quantity of unavailable carbohydrates the higher the energy value of feces voided. This has significance, as in lower income countries foodstuffs may often have more unavailable carbohydrates, therefore, feces of subjects in lower income countries may have fecal energy values higher than the values presented in this study suggest. As a guideline for calorific values fecal dry mass can be used as an estimate for energy losses in feces (reflecting unavailable carbohydrate intake) and energy adsorption by the body is correlated significantly with fecal dry weights (−0.911) (Calloway and Kretsch, 1978).

The high TS concentration of feces gives a good case for the source separation of feces and urine as the addition of urine could add the further problem of dewatering and could resultantly increase costs of thermal treatment processes. Nevertheless a sizeable proportion of urine solids are produced by humans (59 g/cap/day) and the calorific value of urine (1701 kcal/kg) could contribute to energy production if efficient dewatering technologies were available.

Other factors that may be significant for thermal process regard the potential emissions from any thermal treatment process. Levels of sulfur are low in feces but slightly higher levels are observed in the urine fraction, this could be significant as sulfur in oxygen starved conditions is reacted in the form H_2_S (Kang et al., 2011).

## 5. CONCLUSIONS

This review aimed to characterize feces and urine and determine the extent and causes of variation seen and its subsequent impact on technologies treating feces and urine as a fresh waste stream. Table 12 provides a summary of the key criteria and values that will assist in not only the operation of existing OSS systems but will help advance research and development into new OSS technologies.

The generation rate of feces and urine shows significant variation across a wide range of studies presenting difficulties assigning standard design values for treatment technology processes. The values presented are based upon a large database of values from studies worldwide. The median generation rate of feces has been calculated at 128 g/cap/day wet mass and 29 g/cap/day dry mass; however, caution should remain when using these central tendency figures as the data sets were highly skewed. The largest factor leading to variability in fecal mass is the indigestible fiber content of dietary intake; this explains the reason why fecal wet mass values were increased by a factor of 2 in low income countries. A urine generation rate of 1.42 L/cap/day was recorded with the water balance of the body highlighted as the main cause of variation in volume.

Variation in the chemical and physical composition of feces and urine was widespread throughout the study; this means that technology developments must be robust and flexible in order to deal with this uncertainty. It can be concluded however that the composition of feces and urine is highly dependent on the dietary intake of subjects. The predominant factor leading to variation in key parameters in feces was the dietary intake of non-degradable fiber which was shown to impact production rate, stool frequency, TS, fat, protein, and the energy value of feces. In the urine fraction, protein intake was one of the key factors leading to variation in urea concentration as well as impacting concentrations of P, K, and Ca in urine.

Biological treatment processes are likely to be effective at treating feces as a waste stream and a large proportion of the feces are likely to digest readily. However, high non-degradable fiber content of feces may reduce digestibility and with a combined waste stream of feces and urine the anaerobic digestion process may be limited with potential problems such as ammonia toxicity. Technologies based on separation will predominantly be impacted by the variation in TS concentration as well as fluctuating levels of protein and fat found within the feces. Chemical processes will be largely influenced by variation in the diet consumed by subjects, leading to fluctuations in nitrogen and phosphorus loads which could be influential on pH levels, precipitation, and nutrient recovery. Thermal treatment processes will similarly be most influenced by variation in TS as well as the energy content of these solids, once again the intake of fiber proved most influential in predicting these factors.

The source separation of feces and urine could prove beneficial for biological treatment such as anaerobic digestion where large urea concentrations in the urine stream could prove problematic and cause ammonia toxicity. Similarly, the separation of the two streams could increase the efficiency of the dewatering process and make thermal processes increasingly attractive. In addition to this the largest proportion of nutrients (e.g., N, P, and K) are found within the urine fraction making nutrient recovery from urine more attractive from this more easily accessible stream. It is therefore evident that source separation could be beneficial to many treatment technologies.

This study has illustrated that there is significant variation in both the production values as well as the physicochemical composition of feces and urine. Therefore, there are limitations in using standard design values in the development of treatment technology. Consequently it is important that treatment technology is robust and flexible enough to deal with the variation exposed. It is however possible to make more appropriate decisions about values of production and composition through the assessment of a target population's diet. Through this a range of dietary factors can be assessed in order to make more informed decisions about design values that specifically target individual populations. Additional data, especially information regarding how the structure of feces changes over time, would be of further benefit to technology development but there is nevertheless no shortage of data regarding the production and composition of feces and urine.

## FUNDING

The authors gratefully acknowledge financial support from EPSRC for their funding of the “Transforming Waste” project, grant number EP/J00538X/1.
